# Effective Peroxidase-Like Activity of Co-Aminoclay [CoAC] and Its Application for Glucose Detection

**DOI:** 10.3390/s18020457

**Published:** 2018-02-03

**Authors:** Han Pill Song, Yongil Lee, Vu Khac Hoang Bui, You-Kwon Oh, Hyun Gyu Park, Moon Il Kim, Young-Chul Lee

**Affiliations:** 1 Department of BioNano Technology, Gachon University, 1342 Seongnamdae-ro, Sujeong-gu, Seongnam-Si, Gyeonggi-do 13120, Korea; hanpilll@naver.com (H.P.S.); hoangvu210190@gmail.com (V.K.H.B.); 2 Korea Railroad Research Institute (KRRI), 176 Cheoldobakmulkwan-ro, Uiwang-si, Gyeonggi-do 16105, Korea; freego83@krri.re; 3 Department of Mechanical Engineering, Sungkyunkwan University, Suwon, Gyeonggi-do 16419, Korea; 4 School of Chemical and Biomolecular Engineering, Pusan National University, Busan 46241, Korea; youkwan@pusan.ac.kr; 5 Department of Chemical and Biomolecular Engineering (BK21+ Program), Korea Advanced Institute of Science and Technology (KAIST), 291 Daehak-ro, Yuseong-gu, Daejeon 34141, Korea; hgpark@kaist.ac.kr

**Keywords:** Co-aminoclay [CoAC], peroxidase mimetic, fluorescent biosensor, colorimetric sensor, glucose detection

## Abstract

In this study, we describe a novel peroxidase-like activity of Co-aminoclay [CoAC] present at pH ~5.0 and its application to fluorescent biosensor for the determination of H_2_O_2_ and glucose. It is synthesized with aminoclays (ACs) entrapping cationic metals such as Fe, Cu, Al, Co., Ce, Ni, Mn, and Zn to find enzyme mimicking ACs by sol–gel ambient conditions. Through the screening of catalytic activities by the typical colorimetric reaction employing 2,2′-azino-bis(3-ethylbenzo-thiazoline-6-sulfonic acid)diammonium salt (ABTS) as a substrate with or without H_2_O_2_, Fe, Cu, and CoACs are found to exhibit peroxidase-like activity, as well as oxidase-like activity was observed from Ce and MnACs. Among them, CoAC shows exceptionally high peroxidase-like activity, presumably due to its ability to induce electron transfer between substrates and H_2_O_2_. CoAC is then used to catalyze the oxidation of Amplex^®^ UltraRed (AUR) into a fluorescent end product, which enables a sensitive fluorescent detection of H_2_O_2_. Moreover, a highly sensitive and selective glucose biosensing strategy is developed, based on enzyme cascade reaction between glucose oxidase (GOx) and CoAC. Using this strategy, a highly linear fluorescence enhancement is verified when the concentration of glucose is increased in a wide range from 10 μM to 1 mM with a lower detection limit of 5 μM. The practical diagnostic capability of the assay system is also verified by its use to detect glucose in human blood serum. Based on these results, it is anticipated that CoAC can serve as potent peroxidase mimetics for the detection of clinically important target molecules.

## 1. Introduction

Over and beyond the various organic [1] and inorganic [2] nanoparticles (NPs), many hybrid organic-inorganic [3] NPs have been extensively researched in biomedical, environmental, and energy applications for their size, shape, charge, and surface chemistry including diverse functionalities. Specific organic-functional groups in many hybrid NPs offer usually unique properties in terms of the accessibility and bioactivity of targeting cells or biomolecules in bionanotechnology without some post-functionalization in nanotechnology [4,5]. 

One candidate of organic-inorganic NPs with covalent-bonded primary amines, namely 3-aminopropyl-functionalized magnesium phyllosilicate (i.e., Mg-aminoclay [MgAC], formulated as [H_2_N(CH_2_)_3_]_8_Si_8_Mg_6_O_12_(OH)_4_) was developed by one-pot sol–gel reaction under ambient conditions by Mann et al. [6,7] showing unique interactions of organic-pendants with cell or other molecules [8,9] in biomedical fields, as well as with heavy metals [10] in environmental applications. This aminoclay structure is composed of tetrahedral brucite (MgO) in the middle, sandwiched by octahedral silica (SiO_2_) as the unit structure in the vertical direction (i.e., 2:1 trioctahedral clay) and a repeated tetrahedral/octahedral structure in pairs, known as the 1:1 dioctahedral structure. Diverse high-density primary amines (-(CH_2_)_3_NH_2_) in octahedral structures have been coined aminoclays (ACs) [11] according to the cationic metals used in their preparation [12,13].

Recently, the organo-building blocks of Mg and CaACs were tested for possible use as drug-delivery carriers, and were found to result in neither cytotoxicity nor inflammation [14]. Further, the protonated clusters of MgAC with positively charged zeta potential in the wide pH range of 2.0–12.0 were previously reported [15] as well as Cy 5.0 conjugation with organo-building blocks in delaminated MgAC was investigated for the biodistribution and elimination pathways in in vivo mice [16]. The results showed fast elimination or excretion of MgAC in mice after oral or intravenous injection, respectively, without toxicity. With the exceptions of transparent Mg and CaACs in aqueous solutions, other colored aminoclays have not been tested for cytotoxicity to determine the feasibility of their use in biomedical applications. Importantly, the lack of or weak fluorescent-emission intensity of aminoclays has driven research to explore fluorescent imaging for promising drug-delivery-carrier and simultaneous bioimaging applications in diagnostics and therapeutics.

Recently, many kinds of nanostructures have been investigated as enzyme mimetics, which have intrinsic enzyme-like activities. Their catalytic activities were generally recognized in the cationic metal positions, which consist of the ACs frameworks. Among them, FeAC, which was reported by our group, have gathered intense attention as peroxidase mimetics due to their unique advantageous features such as high peroxidase-like activity, facile synthesis and surface-functionalization, high dispersity in aqueous buffer solution, and stability [17]. They have been successfully utilized to colorimetrically detect lung cancer cells after conjugation with folic acid. The promising application results have prompted us to investigate other types of aminoclays using other cationic metals, which presumably show oxidoreductase activity. Herein, we synthesized aminoclays containing diverse cationic metals such as Fe, Cu, Al, Co., Ce, Ni, Mn, and Zn to find new enzyme mimicking ACs. Through the screening of catalytic activity by the typical colorimetric reaction, we found that CoAC has significantly high peroxidase-like activity and applied them to develop fluorescent biosensor for the sensitive determination of H_2_O_2_ and glucose concentrations. Analytical features of the new detection system such as selectivity, sensitivity, and precision were also investigated.

## 2. Materials and Methods 

### 2.1. Preparation of Eight Types of Aminoclays

According to the literature, eight types of ACs were synthesized, based on magnesium-aminoclay [18]. Briefly, respective 8.4 g of FeCl_3_•6H_2_O [17], CuCl_2_•2H_2_O [19], AlCl_3_•7H_2_O [20], CoCl_2_•6H_2_O [21], CeCl_3_•7H_2_O [22], NiCl_2_•6H_2_O [23], MnCl_2_•4H_2_O [19], and ZnCl_2_ [24], (Sigma-Aldrich, St. Louis, MO, USA), were utilized to dissolve in a 500-mL glass beaker containing 200 mL ethanol solution for 10 min magnetic stirring. In case of exceptional anhydrous ZnCl_2_, 1 mL of double distilled water was added to induce hydrolysis of the organosilane layer. Thirteen mL of 3-aminopropyltriethoxysilane (APTS, Sigma-Aldrich, USA) was subsequently added drop-wise into prepared solution. After 5 min mixing, a slurry was immediately produced. For a further 12 h reaction in production, as-prepared ACs were centrifuged at 6000× *g* for 10 min. The harvested ACs were dried at 60 °C for 24 h in an oven and powdered by pestle and mortar before characterization and investigated their potential as glucose biosensor.

### 2.2. Characterizations of As-Prepared Aminoclays

For visual observation of AC dispersed in double distilled water (2.5 mg/mL) by 5 min bath sonication, field emission transmission electron microscopy (FE-TEM, Tecnai F20, Philips, Amsterdam, The Netherlands) microphotographs [16]. In order to confirm the crystalline structure or not in as-prepared ACs or impurities, power X-ray diffraction (XRD, D/MAX-2500, Rigaku, 40 kV and 300 mA) patterns were examined, focusing on ranging from 3 to 70° with increment in 0.01. With FT-IR spectrophotometer (FT-IR 4100, Jasco, Tokyo, Japan) [25], it was determined organic functional groups in aminoclays, after preparation of sample in which it was consisted of 90 wt % KBr and 10 wt % respective AC by a pellet mode [16]. 

### 2.3. Screening of Catalytic Activities of As-Prepared Aminoclays

Peroxidase or oxidase-like activities of as-prepared ACs were screened by performing the representative catalytic oxidation of the colorimetric substrate, ABTS, with or without H_2_O_2_, respectively. In a typical screening for peroxidase-like activity, a solution containing 50 μL of ABTS (50 mM), 50 μL of aminoclays (2 mg/mL), and 50 μL of H_2_O_2_ (100 mM) in 350 μL of tris-acetate buffer (0.1 M at various pHs) was incubated for 5 min at room temperature. After the reaction, the ACs was removed from the reaction solution by centrifugation at 10,000× *g* for 3 min and the supernatant was used to obtain images representing the progress of the reaction. The absorbance intensity of the solution was also measured after 5 times dilution of the reaction solution. Spectrophotometric measurements were carried out in a scanning mode or at 417 nm using a microplate reader (Synergy H1, BioTek, Winooski, VT, USA). 

### 2.4. Fluorometric Detection for H_2_O_2_ Using Co-Aminoclay

The detection of H_2_O_2_ concentration was performed in a black 96-well plate as follows: a reaction buffer solution (140 μL, 0.1 M Tris-acetate, pH 8) containing CoAC (20 μL, 0.1 mg/mL), AUR (20 μL, 500 nM), and H_2_O_2_ at various concentrations were incubated at RT for 5 min. After the reaction, the CoAC was separated from the mixture by centrifugation at 10,000× *g* for 3 min, and the supernatant were further analyzed by measuring the fluorescence with excitation and emission wavelength of 530 nm and 580 nm or in a scanning mode, respectively, using a microplate reader (Synergy H1, BioTek, Winooski, VT, USA). The fluorescent images after the reaction were also obtained by utilizing a fluorescence image analyzer (Image Station 4000mm Pro, Kodak, Stamford, CT, USA). 

### 2.5. Fluorescent Determination of Glucose Using Co-Aminoclay and Glucose Oxidase

The determination of glucose concentration was performed in a black 96-well plate as follows: A PBS buffer solution (10 mM, pH 7.4) containing GOx (10 μL, 10 mg/mL) and glucose at different concentrations (40 μL, 10 μM–20 mM) was incubated at 37 °C for 20 min. Subsequently, another reaction buffer (110 μL, 0.1 M Tris-acetate, pH 8) containing CoAC (20 μL, 0.1 mg/mL) and AUR (20 μL, 500 nM) were added into each well and incubated at room temperature for 5 min. After the reaction, the CoAC was separated and other detection procedures were the same as previously described. For selectivity test, other carbohydrates such as maltose, fructose, lactose, and sucrose were used instead of glucose. 

### 2.6. Glucose Detection in Human Serum

Initial glucose concentration in human serum was first determined by glucose assay kit (Sigma-Aldrich) and then a predetermined amount of glucose was further added into human serum to make spiked samples, which represented normal, boundary, and high level of blood glucose. Finally, glucose concentration of each spiked sample (40 μL, 50-fold dilution) was determined by the same procedures described above.

## 3. Results and Discussion

### 3.1. TEM Observation of Eight Types of Aminoclays

**Figure 1:**
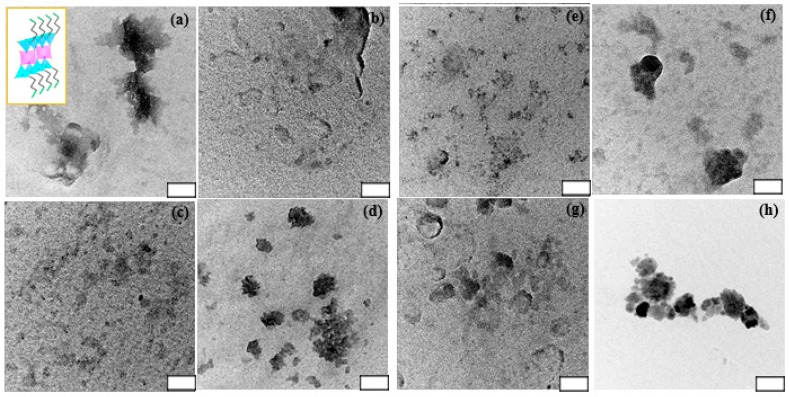
Electron transmission microscopy (TEM) images of FeAC (**a**); CuAC (**b**); CoAC (**c**); ZnAC (**d**); AlAC (**e**); NiAC (**f**); CeAC (**g**); and MnAC (**h**) dispersed in phosphate buffered saline (PBS buffer; 0.01 M and pH 7.2) at 1.0 mg/mL. Inset in (a) shows schematic representation of approximate unit structure of ACs. Note that white box scale is 50 nm.

For eight types of ACs, it showed water-soluble or water-dispersible AC NPs by delaminated clay sheets, which is associated with protonated amine (-NH_3_^+^) formation with a high density. Figure 1 represented transmission electron microscopy (TEM) images of eight types of ACs. As previously reported microphotographs [10], ACs shows the diameter sizes ranging from 20 nm–200 nm, with a similar morphology like sheet-like and layered structures with different contrast, according to stacking clay sheet layers [18]. The distribution size of ACs is displayed in Figure S1. The polydispersity index (PDI) of ACs is ranging from 0.213–0.444 (Table S1). It is matched with water-soluble or water-dispersible properties as mentioned above. Additionally, at the high magnification images, it is rarely observed lattice fringe, indicating amorphous (partially crystalline) structures, exception of CuAC and MnAC. Inset in Figure 1a showed a side view of ideal unit structure of AC where a pink-colored octahedral sheet containing respective cationic metals with sandwiched tetrahedral silica sheets in sky-blue color as well as green-colored pendents are –NH_2_ functionalities. The chemical formula of AC may be supplanted with a replacement of cationic metal, based on the reported result of MgAC, i.e., [H_2_N(CH_2_)_3_]_8_Si_8_Mg_6_O_12_(OH)_4_ [7]. From a focus ion-beam scanning electron microscope (FIB-SEM) (Figure S2) and scanning electron microscope (SEM) (Figure S3), CoAC and MnAC exhibited aggregated form, but in a larger and more dense structure compared with other ACs, with more porosity. The difference in this observation may lead to the difference in accessibility.

### 3.2. XRD Patterns and FT-IR Spectra of Eight Types of Aminoclays

**Figure 2:**
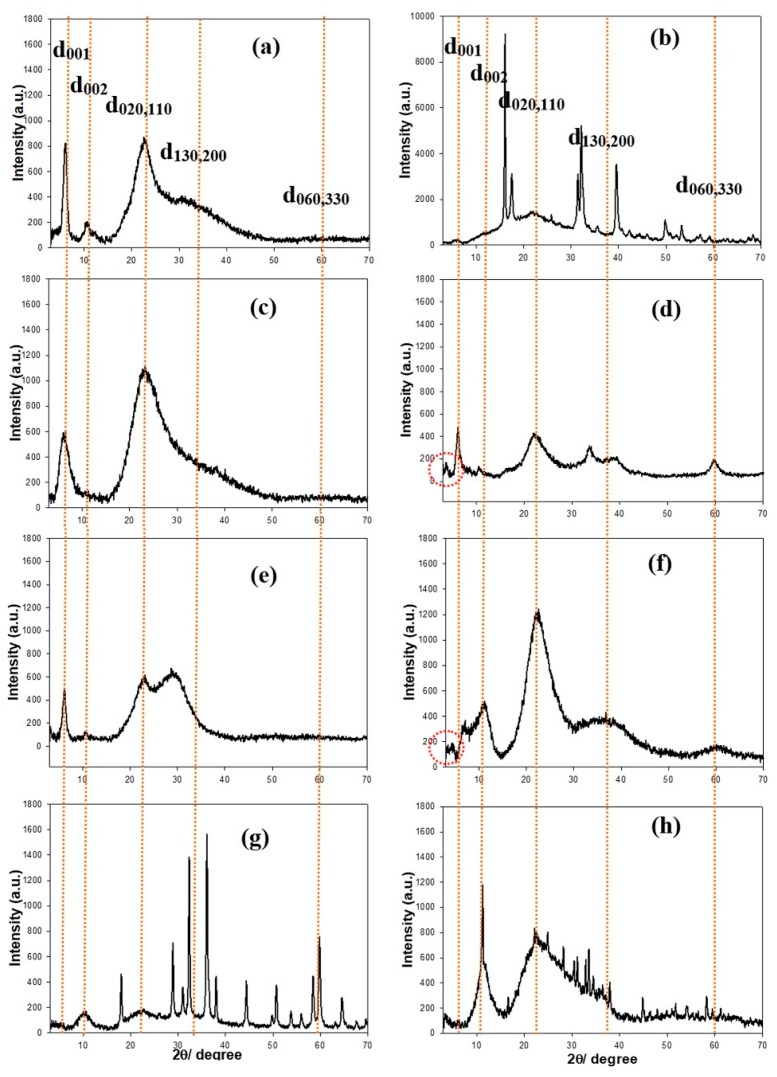
Powder X-ray diffraction (XRD) patterns of FeAC (**a**); CuAC (**b**); CoAC (**c**); ZnAC (**d**); AlAC (**e**); NiAC (**f**); CeAC (**g**); and MnAC (**h**). Note that orange-colored dotted lines are marked by eye guide.

For identification and to check for the crystalline/amorphous phases in materials, the powder X-ray diffraction (XRD) patterns of aminoclays were examined (Figure 2). The regular distance in the at d_001_ was calculated to ~14.0 Å at 2θ = 6.0° in FeAC, and in the broad peaks at in-plane reflection, the distances were ~7.8, ~3.9, and ~2.8 Å at 2θ = 11.3, 22.7, and 32.3°, respectively without distinct peak at ~2θ = 59° (Figure 2a), finding that it is matched with 2:1 dioctahedral phyllosilicate, corresponding to previous data [26]. Similarly, both AlAC and CeAC showed 2:1 dioctahedral phyllosilicate structure (Figure 2e,g). However, AlAC represented the peak of d_130,200_ was very weak intensity while CeAC displayed that of d_130,200_ was shifted to smaller angle at ~2θ = 30°. Particularly, XRD patterns of CuAC and MnAC showed similar sharp peaks, in line with previous data, respectively (Figure 2b,h). On the other hands, both CoAC and NiAC exhibited typical 2:1 trioctahedral phyllosilicate structures (Figure 2c,f) [21,23]. Interestingly, it was found that additional peaks at 2θ = 3.55° and 4.14° with mesolar-structured 24.87 Å and 21.33 Å in CoAC and NiAC respectively. In case of ZnAC (Figure 2d), it presented similar FeAC or AlAC structure in general and specifically showed sharp peaks, looking as those of CuAC or MnAC [19].

**Figure 3:**
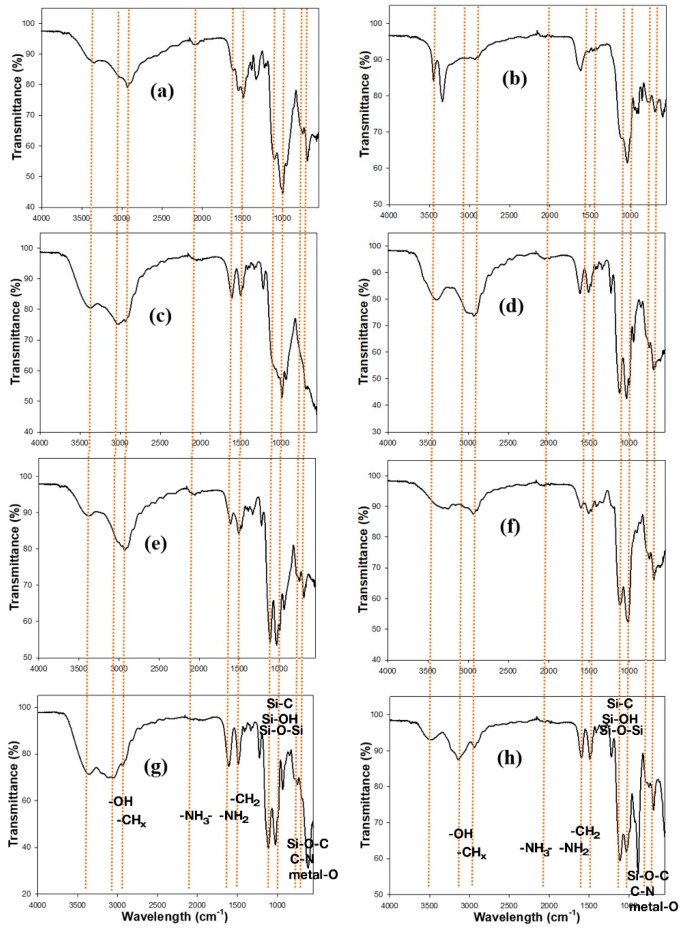
Fourier transform infrared (FT-IR) spectra of FeAC (**a**); CuAC (**b**); CoAC (**c**); ZnAC (**d**); AlAC (**e**); NiAC (**f**); CeAC (**g**); and MnAC (**h**) at by KBr pellet mode.

The covalent bindings in organofunctional groups in aminoclays were recorded with the Fourier transform infrared (FT-IR) spectra (Figure 3). In general, the FT-IR spectra peaks were assigned as follows: -OH (3300 cm^−1^), -CH_x_ (2920 cm^−1^), -NH_3_^+^ (2020 cm^−1^), -NH_2_ (1609 cm^−1^), -CH_2_ (1487 cm^−1^), Si-C (1120 cm^−1^), Si-OH (1034 cm^−1^), Si-O-Si (1014 cm^−1^), Si-O-C (770 cm^−1^), C-N (680 cm^−1^), metal-O-Si (~650 cm^−1^), and metal-O (<600 cm^−1^), indicating that organic pendants in inorganic framework are successfully covalented onto each metal species [20].

### 3.3. Screening of Peroxidase or Oxidase-Like Activity of Diverse Aminoclays

**Figure 4:**
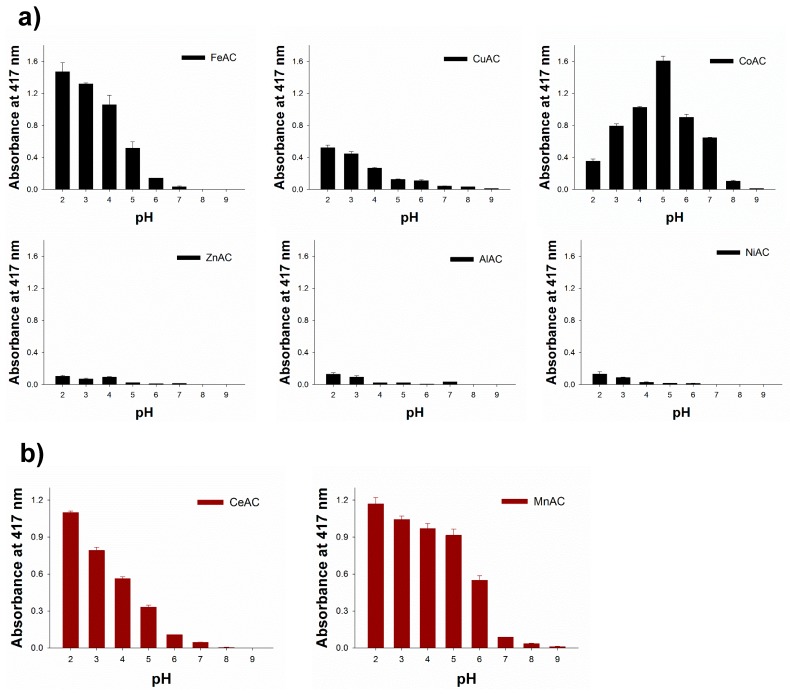
Catalytic screening for (**a**) peroxidase and (**b**) oxidase-like activity of several ACs by the oxidation of ABTS in the presence or absence of H_2_O_2_, respectively.

To find novel peroxidase or oxidase mimetics among the synthesized cationic metal-containing ACs, the aminoclays along with FeAC, which was already proven to be peroxidase mimetics by our group [17], were examined for their peroxidase or oxidase-like activities to promote the oxidation of ABTS in the presence or absence of H_2_O_2_. Through the typical reactions performed in a range of pHs, Fe, Cu, and CoACs showed peroxidase-like activity, which catalyzed the oxidation of ABTS only in the presence of H_2_O_2_. At the same time, Ce and MnACs showed oxidase-like activity catalyzing the oxidation of ABTS even without H_2_O_2_ (Figure 4). There were no significant color change or absorbance enhancement from Al, Ni, and ZnACs. Among the enzyme mimicking ACs, CoAC exhibited a significant amount of catalytic activity at around pH 5, which make them highly applicable in diverse peroxidase-mediated applications. It is presumed that the catalytic activity of CoAC may have arisen from the capability of electron transfer of cobalt ions between substrates and H_2_O_2_ to produce hydroxyl radicals, which are directly involved in the oxidation of employed substrate, ABTS [27]. We also confirmed that there is negligible activity with the aqueous solutions containing Co. ions (10%, 20%, and 30% compared to the concentrations of CoAC (Figure S4), indicating that free Co. ions cannot catalyze the oxidation of ABTS (Figure S5). CoAC was also very stable at various pH conditions, making its incubation suitable at various pH environments (Figure S6). 

### 3.4. Investigation for Peroxidase-Like Activity of Co-Aminoclay for the Determination of H_2_O_2_

**Figure 5:**
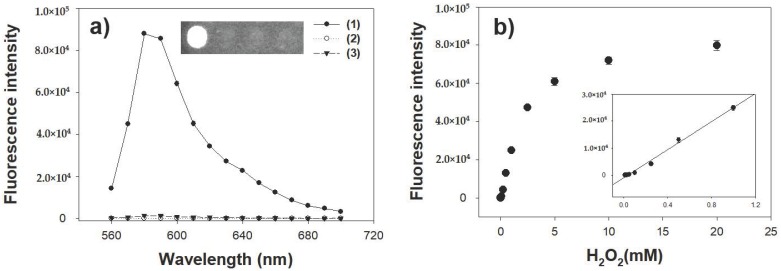
(**a**) Fluorescence spectra and the corresponding images for the CoAC-based detection of H_2_O_2_. Sample specifications: (1) CoAC with H_2_O_2_; (2) CoAC without H_2_O_2_; and (3) H_2_O_2_ without CoAC; (**b**) A dose-response curve for H_2_O_2_ detection by CoAC. Inset represents the corresponding linear calibration plot. The error bars represent standard deviations derived from three independent measurements.

Since the peroxidase-like activity of CoAC to oxidize employed substrate is promoted by H_2_O_2_, the ACs were then employed to detect H_2_O_2_ dissolved in sample solution. To achieve higher sensitivity, another peroxidase-mediated substrate, AUR, which is also widely used for the peroxidase-mediated reaction at around neutral pH condition, was employed in this assay system [28]. Through the typical reaction condition described in Experimental section, fluorescence intensity by the oxidation of AUR was specifically observed with both CoAC and H_2_O_2_ while other control cases exhibited negligible fluorescence intensities, indicating that CoAC displayed high peroxidase activity with AUR as a substrate (Figure 5a). The fluorescence intensity upon oxidation of AUR sharply increased with increasing H_2_O_2_ concentrations up to approximately 10 mM (Figure 5b). As shown in the inset of Figure 5b, a linear relationship (R^2^ = 0.99) between fluorescence intensity and H_2_O_2_ concentration in a range of 10 μM to 1 mM with a low detection limit of 1 μM was obtained, which indicates that CoAC-based system could be practically used in analytical field for the detection of H_2_O_2_. This level of sensitivity is sufficient to enable coupling of CoAC with any oxidase to create a versatile fluorescent sensor [29].

### 3.5. Analytical Capabilities of Co-Aminoclay for the Detection of Glucose

**Figure 6:**
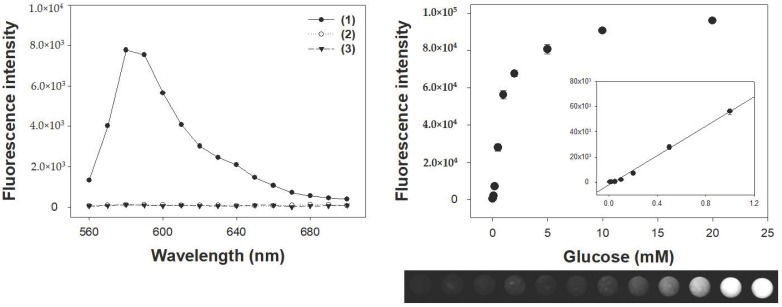
(**a**) Fluorescence spectra for the CoAC-based detection of glucose. Sample specifications: (1) CoAC and GOx with glucose; (2) GOx with glucose in the absence of CoAC; and (3) CoAC and GOx without glucose; (**b**) a dose-response curve for glucose detection and the corresponding images by CoAC-based glucose biosensor. Inset represents the corresponding linear calibration plot. The error bars represent standard deviations derived from three independent measurements.

The feasibility of the glucose biosensor was demonstrated by coupling glucose oxidase (GOx) with CoAC. In the presence of target glucose, GOx catalyzed the oxidation of glucose to generate H_2_O_2_, which should activate CoAC to convert AUR into highly fluorescent product. Through the typical reaction, fluorescence intensity corresponding to the oxidized AUR was specifically observed in the reaction solution containing glucose, GOx, and CoAC altogether, while other control samples did not yield any significant fluorescence intensities, clearly demonstrating that CoAC coupled with GOx displayed specific fluorescence in the presence of target glucose (Figure 6a). From an analysis of dose-response curves using CoAC for glucose detection, the limit of detection for glucose was determined to be as low as 5 μM in the linear range from 10 μM to 1 mM (Figure 6b). These limit of detection (LOD) and linear range values are among the best results of those recently reported, describing the various enzyme mimetics-based colorimetric measurements of glucose [30,31].

**Figure 7:**
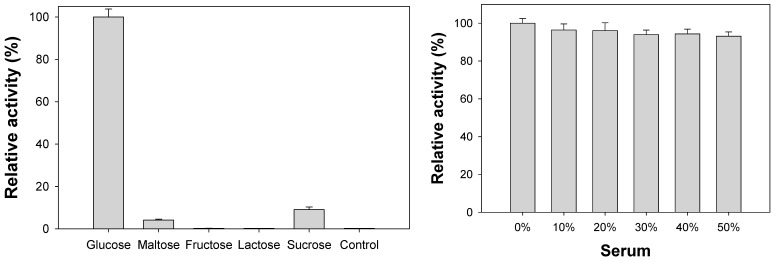
(**a**) Selectivity of CoAC-based biosensor for the determination of target glucose. One mM glucose was used for the experiment while 10 mM of other carbohydrates were used as negative controls; (**b**) clinical applicability of CoAC-based biosensor for the determination of glucose dissolved in increasing amounts of human serum.

We then examined the selectivity of CoAC-based biosensor for the fluorescent determination of glucose. Through the typical reaction for glucose detection, glucose molecules are successfully detected by the generation of distinct fluorescence intensity (Figure 7a). On the other hand, no significant fluorescence intensity is observed in the negative control samples where carbohydrate molecules similar to the target glucose are used even at tenfold higher concentrations. These results demonstrate that a specific fluorescent reaction of only the target glucose is promoted by the CoAC-based system. Furthermore, we evaluated the clinical applicability of CoAC-based glucose biosensor by determining glucose concentrations in human serum samples. As shown in Figure 7b, the fluorescent responses of CoAC-based biosensor were measured with 1 mM glucose dissolved with different amounts of human serum. The results show that the presence of serum had negligible effect and did not induce any significant changes (<7%) in fluorescence responses, demonstrating the CoAC-based glucose biosensing platform could represent a practical analytical system capable of diagnosing high level of glucose (hyperglycemia) [32] in complex clinical samples like human blood serum.

### 3.6. Diagnosis of High Level of Glucose (Hyperglycemia) Using Blood Serum Samples

Finally, this fluorescent glucose assay was employed to diagnose different stages of glucose in real human blood serum, which correspond to the normal, boundary, and high stage of hyperglycemia (normal; ≤5.6 mM, boundary; 5.6–7 mM, and high; >7 mM) [32]. Original amount of glucose in serum sample was first determined by glucose assay kit (Sigma-Aldrich, MO, USA) and then predetermined amount of glucose was further added to make the representative levels. As a result, the serum glucose levels were quantified with excellent precisions yielding CVs in a range of 1.27–5.06% and recovery rates of 99.7–101.0% (Table 1), proving the excellent reproducibility and reliability of the method. These results demonstrate that the CoAC-based assay system should serve as a promising analytical tool to diagnose high level of glucose in a real clinical system.

## 4. Conclusions

In summary, taking into the consideration of practical diagnostics for clinically important target molecules, this work has shown that CoAC demonstrated a promising analytic tool for glucose detection in blood serum samples at pH ~5.0 as peroxidase mimetics in the presence of H_2_O_2_, after screening with FeAC, CuAC, CoAC, ZnAC, AlAC, NiAC, CeAC, and MnAC. CoAC catalyzed the oxidation of AUR to detect fluorescent H_2_O_2_. In the range from 10 µM to 1 mM of glucose, it was verified with highly sensitive and selective biosensing.

## Figures and Tables

**Table 1:** Detection precision of the CoAC-based biosensor for the determination of glucose levels in spiked human serum samples.

	Original Amount (mM)	Added (mM)	Expected (mM)	(mM)	SD	CV (%)	Recovery (%)
Normal	1.46	2.5	3.96	3.95	0.05	1.27	99.75
Boundary		5	6.46	6.52	0.33	5.06	100.93
High		10	11.46	11.96	0.55	4.60	104.44

## Supplementary Materials

The following are available online at http://www.mdpi.com/1424-8220/18/2/457/s1, Figure S1: The size distribution of ACs , Figure S2: Focus ion beam scanning electron microscope (FIB-SEM) images of ACs, Figure S3: Scanning electron microscope (SEM) images of FeAC (a), CuAC (b), CoAC (c), ZnAC (d), NiAC (e), CeAC (f), and MnAC (g), Figure S4: Energy-dispersive X-ray (EDX) data of CoAC, Figure S5: Time-dependent absorbance changes at 417nm. Sample specifications are as follows. (1) CoAC with H_2_O_2_, (2), (3), and (4) represent the reactions containing CoCl_2_ (10%, 20%, and 30% compared to the concentration of CoAC, respectively) with H_2_O_2_, Figure S6: Time-dependent absorbance changes at 417 nm. Sample specifications are as follows. (1) CoAC with H_2_O_2_, (2), (3), (4), (5), and (6) represent the reactions containing supernatant solutions after incubating CoAC for 1 hour in five different buffers (pH 2, 4, 6, 8, and 10 with 0.1 M Tris-acetate, respectively) with H_2_O_2_, Table S1: Polydispersity index (PDI) of ACs.
