# Supplementary material for: Effective Peroxidase-Like Activity of Co-Aminoclay [CoAC] and Its Application for Glucose Detection
Source: Sensors (Basel). 2018 Feb 3;18(2):457. doi: 10.3390/s18020457 (PMC5855466; doi:10.3390/s18020457)
Supplement: Supplementary file 1 [file sensors-18-00457-s001.pdf]

Supporting Information

Table S1. Polydispersity index (PDI) of ACs

Aminoclay	PDI
FeAC	0.253
CuAC	0.247
CoAC	0.231
ZnAC	0.344
NiAC	0.381
CeAC	0.336
MnAC	0.444

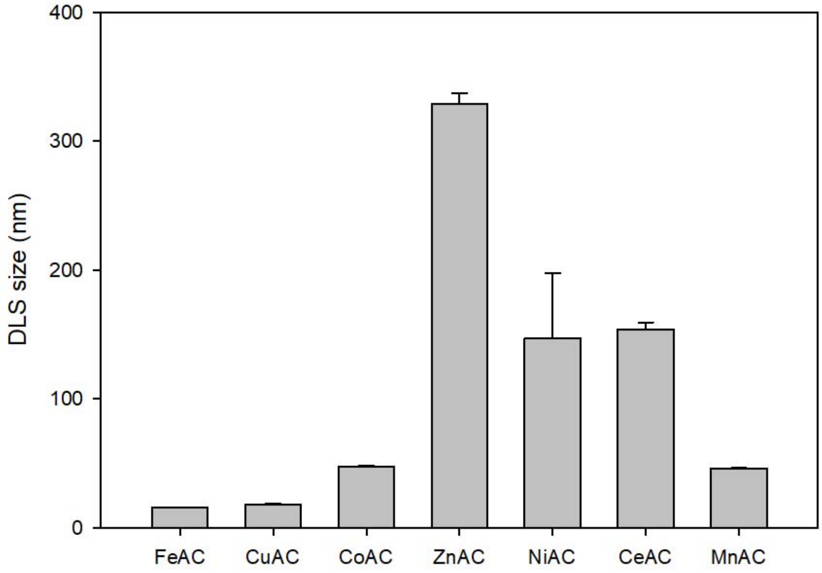

Figure S1. The size distribution of ACs

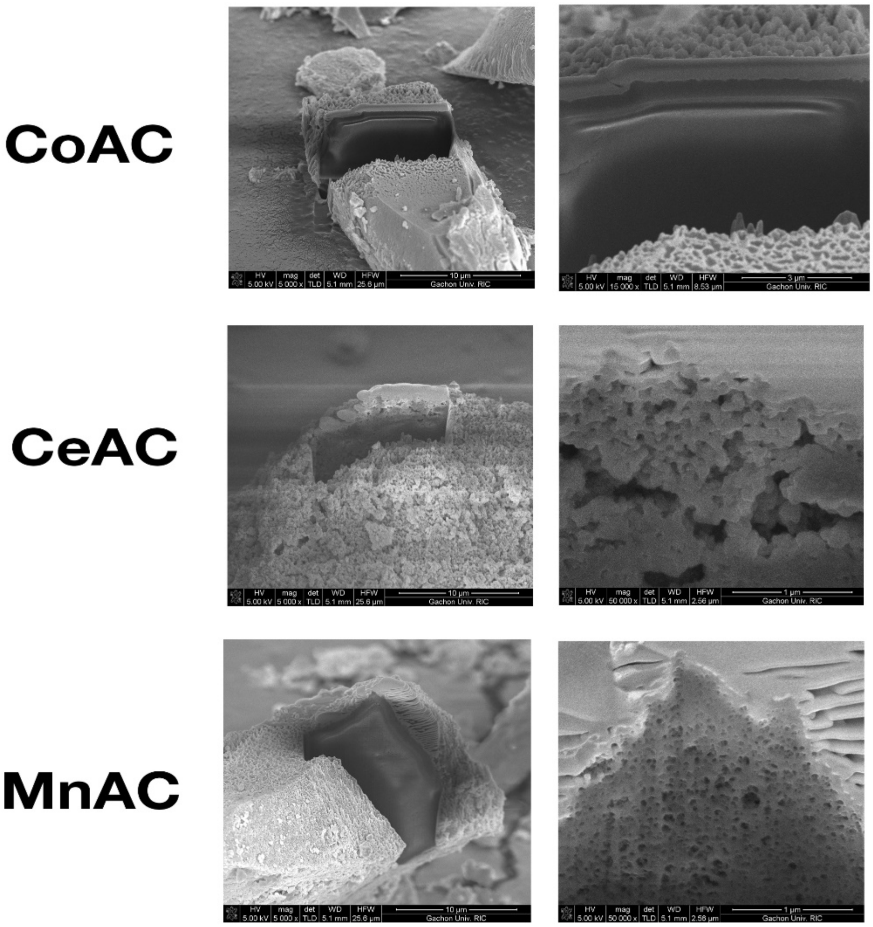

Figure S2. Focus ion beam scanning electron microscope (FIB-SEM) images of ACs

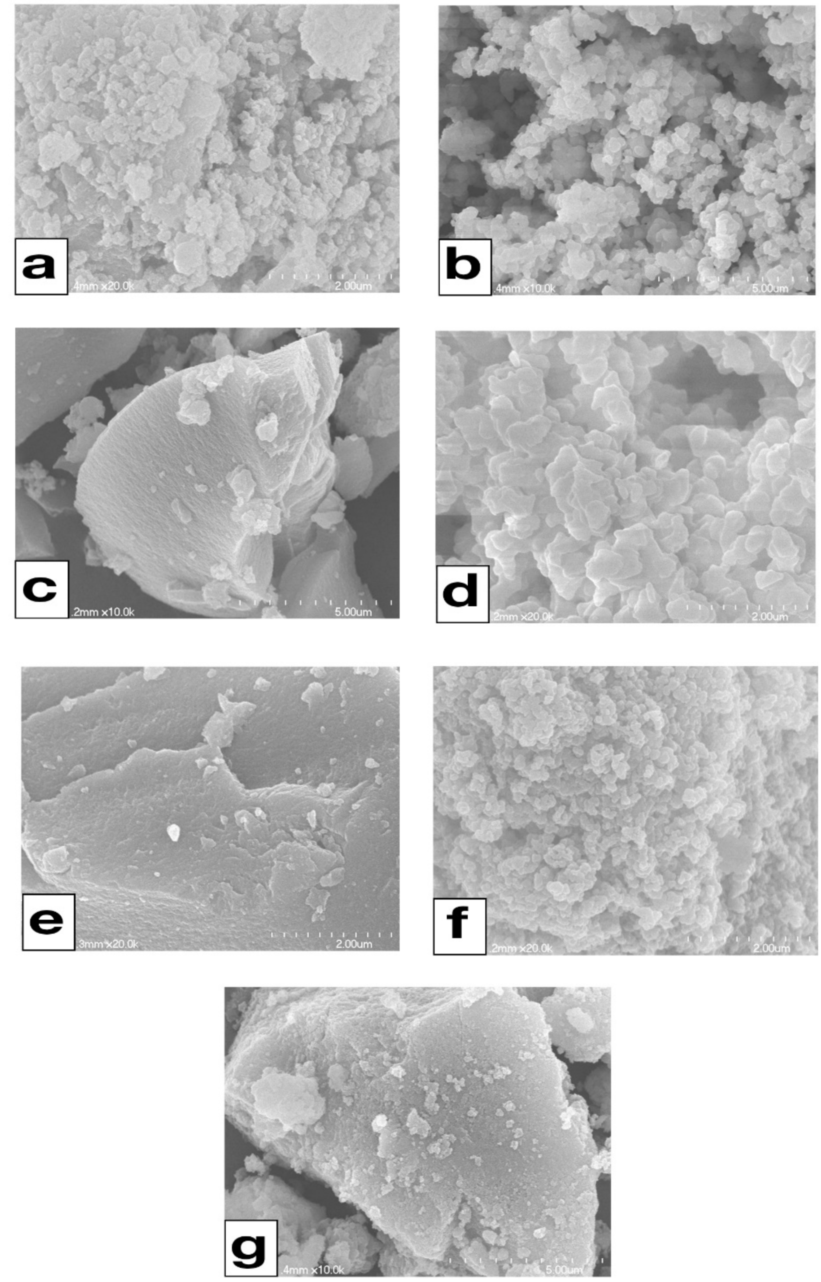

Figure S3. Scanning electron microscope (SEM) images of FeAC (a), CuAC (b), CoAC (c), ZnAC (d), NiAC (e), CeAC (f), and MnAC (g).

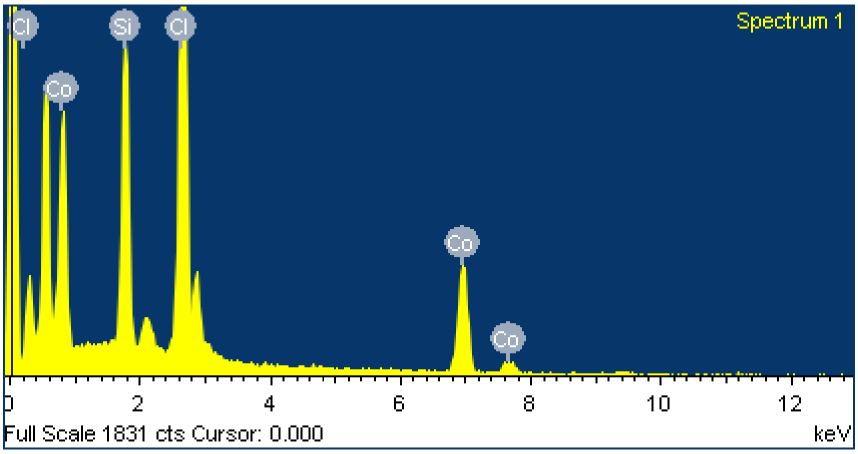

Element	Weight%	Atomic%
Si K	18.10	26.48
Cl K	35.52	41.17
Co K	46.39	32.35
Totals	100.00	

Figure S4. Energy-dispersive X-ray (EDX) data of CoAC

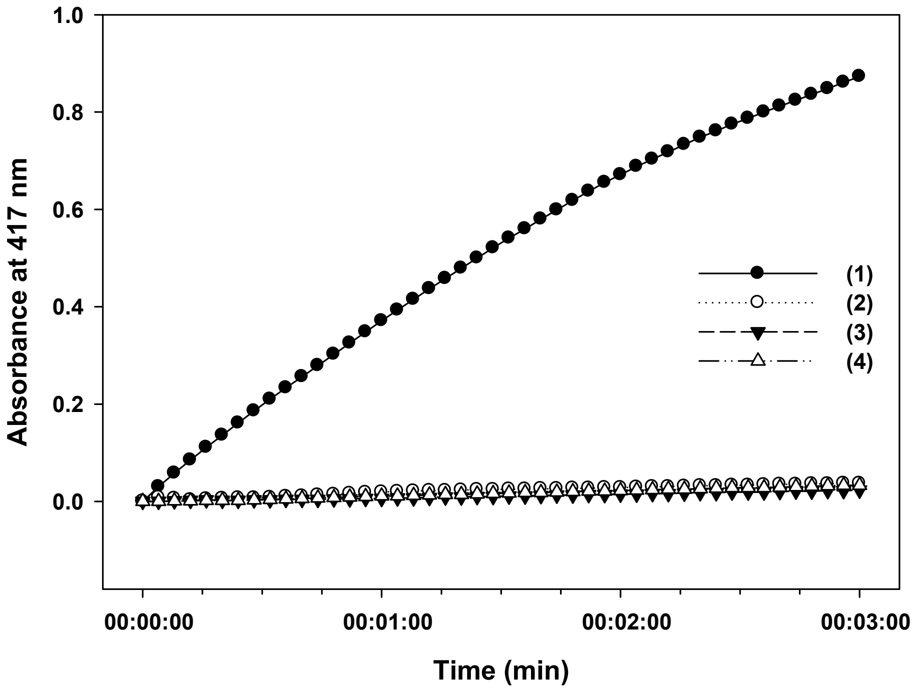

Figure S5. Time-dependent absorbance changes at 417nm. Sample specifications are as follows. 1) CoAC with H_2O_2, 2), 3), and 4) represent the reactions containing $CoCl_2$ (10%, 20%, and 30% compared to the concentration of CoAC, respectively) with H_2O_2.

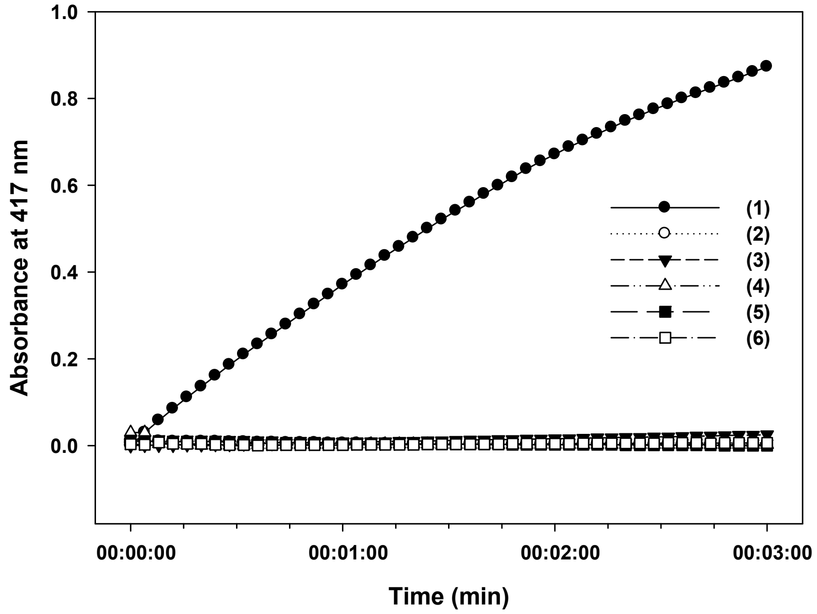

Figure S6. Time-dependent absorbance changes at 417 nm. Sample specifications are as follows. 1) CoAC with H_2O_2, 2), 3), 4), 5), and 6) represent the reactions containing supernatant solutions after incubating CoAC for 1 hour in five different buffers (pH 2, 4, 6, 8, and 10 with 0.1 M Tris-acetate, respectively) with H_2O_2.
